# Healthcare related barriers and enablers for weight management among pregnant women with overweight and obesity: a rapid scoping review

**DOI:** 10.1186/s12884-025-07262-3

**Published:** 2025-03-07

**Authors:** Vidanka Vasilevski, Alemayehu Mekonnen, Anna Peeters, Anna Chapman, Shaan Naughton, Eva Yuen, Jaithri Ananthapavan, Elizabeth Holmes-Truscott, Jane Willcox, Kristen Graham, Linda Sweet

**Affiliations:** 1 School of Nursing and Midwifery, Centre for Quality and Patient Safety Research, Deakin University, 221 Burwood Hwy, Burwood, 3125 VIC Australia; 2 Western Health, Melbourne, Australia; 3 Institute for Health Transformation, Deakin University, Melbourne, Australia; 4 Deakin Health Economics, Deakin University, Melbourne, Australia; 5 Global Centre for Preventive Health and Nutrition, School of Health and Social Development, Deakin University, Melbourne, Australia; 6 Australian Centre for Behavioural Research in Diabetes, Melbourne, Australia; 7 Royal Women’s Hospital, Melbourne, Australia; 8 National Centre for Epidemiology and Public Health, Australian National University, Canberra, Australia

**Keywords:** Pregnancy, Weight management, Barriers, Enablers, Obesity, Stigma

## Abstract

**Background:**

Overweight and obesity in pregnancy are associated with health risks for women and babies. Providing effective weight management during pregnancy is necessary to support appropriate gestational weight gain and improve outcomes for women and their infants. This study aimed to synthesise evidence documenting healthcare-related barriers and enablers for weight management among pregnant women with overweight or obesity in English-speaking high-income countries.

**Methods:**

An initial rapid scoping review focusing on the healthcare-related barriers and enablers for weight management in all populations with overweight or obesity was undertaken. Due to the unique weight management needs of pregnant women, this study analysed a sub-set of publications collected in the initial review pertaining to pregnant women. All publication types (except protocols and conference abstracts) were eligible for inclusion. The search was limited to publications from 2010 onwards. Academic and grey literature were identified, screened, and data extracted. Findings were summarised thematically.

**Results:**

The initial review search, including all populations, identified 12,762 unique abstracts, and 181 full-text articles. Of these, 22 focused on pregnant women living with overweight or obesity. A further four articles were identified via citation searches of the included articles. The identified barriers and enablers for pregnancy weight management in healthcare settings fell under three broad themes: (1) access to, and engagement with, weight management advice during pregnancy (2), challenges for providing weight management support in healthcare settings, and (3) healthcare provider confidence in providing weight management advice during pregnancy.

**Conclusion:**

Pregnant women with overweight or obesity are not receiving adequate weight management guidance. Multi-level strategies are needed to ensure pregnant women have access to weight management care that is stigma-free, easily accessible, tailored to their individual needs and fosters positive relationships with healthcare providers.

## Background

Recent statistics from high-income countries such as the United States, Australia, and the United Kingdom show that about two-thirds of women are overweight, and of these, half have obesity [1–3]. Over 50% of these women are of childbearing age (18–44 years) [4], which means more women are becoming pregnant whilst experiencing overweight or obesity. Data over the past two decades suggests that the rates of pregnant women with overweight or obesity has been continually rising [5], with over 50% of women presenting for antenatal care with a Body Mass Index (BMI) in the overweight or obesity ranges [6, 7].

Pregnant women living with overweight or obesity are at an increased risk of perinatal complications such as gestational diabetes, hypertension, pre-eclampsia, and emergency caesarean section [8]. Their infants are also at increased risk of prematurity, large for gestational age, birth-related trauma, hypoglycaemia, neonatal intensive care unit admission [8], and stillbirth [9]. Infants born to mothers with obesity during pregnancy may also experience worse long-term health outcomes, with increased risk (67–88%) of being diagnosed with neurodevelopmental problems [10] and a two-fold risk of developing metabolic disorders in later life [11]. For women with and without pre-pregnancy overweight or obesity, excess gestational weight gain has been associated with future obesity for their infants [12–14]. Women with overweight or obesity prior to pregnancy, are however, more likely to exceed [15] gestational weight gain guidelines [16, 17]. Together, findings demonstrate that pregnancy overweight or obesity is a risk factor for maternal/child morbidity and mortality.

Addressing the increased risk of maternal/child morbidity and mortality due to overweight and obesity during pregnancy is necessary to improve health outcomes, as well as reduce the associated healthcare system burden. While actively trying to achieve reductions in weight is not advisable in pregnancy, preventing excess gestational weight gain has been associated with better perinatal outcomes in women with overweight and obesity, such as reduced rates of preterm birth, small for gestational age infants, and gestational hypertension [18–20]. Systematic reviews of behavioural lifestyle interventions in pregnancy have, however, demonstrated mixed results, with some showing significant reductions in gestational weight gain [21, 22] and others having no effect on gestational weight gain [23]. This variability in outcomes has been explained by different intervention approaches [22] and barriers to intervention engagement experienced by women [23]. Clinicians also report challenges in delivering weight management interventions during pregnancy [24, 25], which limits the support women receive to improve their pregnancy outcomes.

To improve access to pregnancy weight management advice and programs in healthcare settings, a comprehensive understanding of healthcare-related barriers and enablers for weight management in pregnancy is required. The aim of this study was to conduct a rapid scoping review to identify barriers and enablers to effective weight management in healthcare settings for pregnant women living with overweight or obesity, in English-speaking, high-income countries.

## Methods

A larger rapid scoping review was conducted to identify barriers and enablers for effective weight management for people living with overweight and obesity [26]. The current study presents a sub-analysis of studies from the larger scoping review that focussed on pregnant women living with overweight or obesity. This was conducted as weight management in pregnancy is different to other groups, therefore it was important to synthesise the unique barriers and enablers to weight management for this population. A rapid scoping review follows the principles of a systematic review, however, with some simplification of steps to ensure a timely and accurate synthesis of evidence [27]. In a rapid scoping review, risk of bias and quality assessment is not required, and limits can be placed on searches and eligibility criteria to expedite review completion [28]. In the current study, risk of bias and quality assessment was not conducted, and limits were placed on date range, setting, and number of web results searched (first ten pages). A protocol was developed prior to conducting the initial review based on the Preferred Reporting Items for Systematic review and Meta-Analysis Protocols (PRISMA-P) guidelines [29] and the PRISMA extension [30] for Scoping Reviews (PRISMA-ScR) was used as a framework for conducting the rapid scoping review.

### Search strategy

A search for publications was undertaken in four electronic databases that index medical and health-related research: CINAHL, PsycINFO, Medline, and Embase. Additional searches were conducted using Google Scholar and by handsearching papers and relevant government, health, and Non-Government Organisation websites. The search included key terms and synonyms related to overweight and obesity, outcomes (e.g., barriers to effective weight management) and context (e.g., studies from high-income countries). The keywords were hand-picked from the literature during the preliminary literature search. Boolean operators (OR, AND) were used to combine the key concepts and were tailored to each database search to capture relevant studies (for the complete details, see Mekonnen et al. [26]). Search results were then imported into EndNote^®^ [31] to manage article collections and were then transferred to Covidence for independent screening.

### Eligibility criteria

The original rapid scoping review included publications that described barriers and enablers to weight management in healthcare settings for all populations with overweight or obesity [26]. Publications that focussed only on pregnant women from the original review were eligible for inclusion in the present analysis. Publications that included perspectives of women who have been pregnant, healthcare providers, or reviewed strategies to improve access to weight management advice/services during pregnancy were included. Due to the rapid scoping review design, only publications set in English-speaking, high-income countries since January 2010 were eligible. Eligible evidence sources were all article types except conference abstracts and study protocols.

Study screening, data extraction, and data synthesis.

Each publication was independently screened by two reviewers at all stages. Title and abstract screening were initially conducted to exclude clearly ineligible publications, followed by full-text screening to determine eligibility. Conflicts were resolved through discussion or, if agreement could not be reached, with adjudication from a third independent reviewer. At the full-text screening stage, only publications that fully met the inclusion criteria were selected to progress to data extraction. The study team extracted data using an electronic survey form on Qualtrics^®^ [32] including the following items: reference details (e.g., author, year, country), article type, context (e.g., service type/setting), and clinical population group (e.g., pregnant women, children). As extraction included the identification of clinical population groups for each of the papers, those focussed on pregnant populations could be easily identified for synthesis of findings in this review. Data from included studies were coded using NVivo^®^ [33] and thematically analysed by two authors. Two of the authors (VV, KG) independently reviewed each paper and coded content on barriers and enablers to effective weight management. An inductive thematic analysis [34] was conducted to identify patterns and themes within the data. Barriers and enablers to weight management described in the literature were grouped and organised into major themes. Any differences in codes and themes were resolved through discussion between the authors (VV, KG).

## Results

**Fig. 1:**
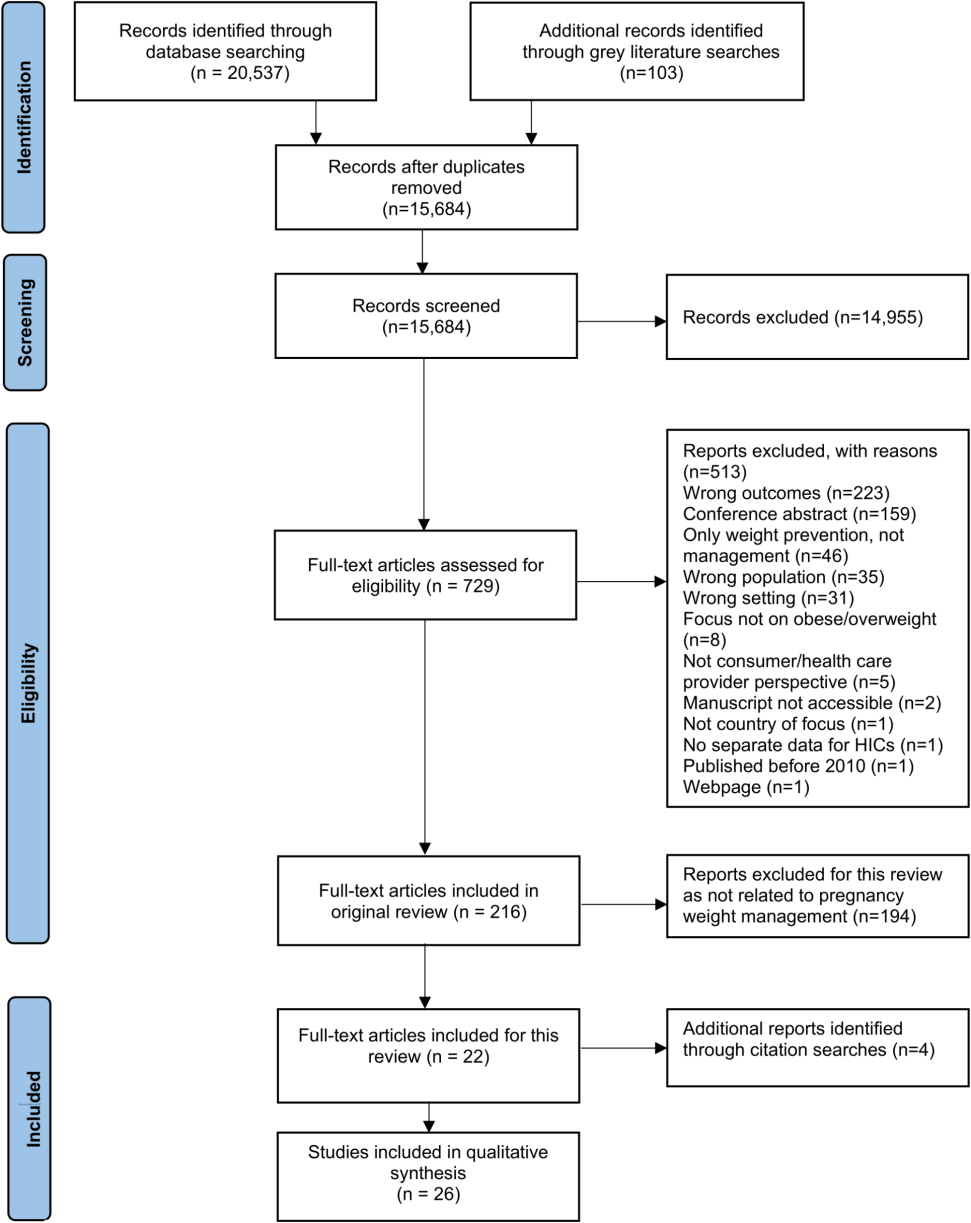
PRISMA flow diagram

The search strategy for the original rapid scoping review identified 20,537 records, of which 15,684 records remained after removal of 4,853 duplicates. Following title and abstract screening, 729 papers were subject to full-text review, with a further 513 papers excluded with reasons. The original scoping review included 216 publications [26]. For the sub-analysis included in the current study, papers not focussed on pregnant populations were excluded (*n* = 194), leaving a total of 22 studies. A further four publications were identified via citation searches of included papers, resulting in a total of 26 studies for inclusion in the current synthesis (Fig. 1).

### Characteristics of included studies

Characteristics of the included studies are summarised in Table 1. Most studies were conducted in the United Kingdom (*n* = 14) and Australia (*n* = 6). A smaller proportion were from the United States of America, the Republic of Ireland, and New Zealand. Two studies (which were systematic reviews) reported on data from a combination of high-income English-speaking countries. Most of the papers were published from 2013. The majority (*n* = 21) of the studies were primary research publications, which employed either qualitative (*n* = 13), mixed methods (*n* = 6), or quantitative designs (*n* = 2). Two papers were discussion pieces, one a PhD thesis [35] and two were systematic reviews, one of which was focussed on the implementation of pregnancy weight management and obesity guidelines [36], and the other on healthcare provider barriers and facilitators to implementation of weight management interventions during pregnancy [37].

**Table 1:** Characteristics of included studies

Authors, year	Country	Article type	Context (i.e., service type/setting)	Healthcare providers included	Clinical population included	Study design, sample size (if applicable)	Data collection type	Major findings	
								Barriers	Enablers
Atkinson S & McNamara PM. 2017	Republic of Ireland	Research paper	Hospital	N/A	Postnatal women	Qualitative, 15 consumers	Interviews	A lack of information & support received from healthcare providers; conflicting information; avoidance of communicating about obesity; language used in conversations	
Davis DL, et al. 2012	Australia	Research paper	Hospital	Midwives	Pregnant Women	Mixed Methods, 17 healthcare providers & 98 consumers	Data audit	Women - reasons for decline: service at unsuitable time/location; lack of childcare to attend service. Midwives: Issue of obesity can be distressing for women to hear during pregnancy; avoiding causing offence; difficulty raising the topic of obesity for midwives who themselves were overweight; lack of transport, childcare and time constraints for women; competing priorities in time-limited consultations; women not concerned about weight status; confidence in providing weight management advice dependant on midwife knowledge.	Midwives: Experience promotes confidence/skills; awareness of service by women; attractive and informative advertising of service promotes maternal request for service.
Dinsdale S, et al. 2016	UK	Research paper	Hospital	N/A	Postnatal Women	Qualitative, 24 consumers	Interviews	Inconsistent communication; not being offered weight management services; referral delays; women feeling judged by healthcare providers.	
Fair, FJ et al. 2022	UK	Research paper	Hospital	N/A	Pregnant women	Qualitative, 13 consumers	Interviews	Lack of specific advice or inundated with too much pregnancy advice; inconsistent advice; lack of understanding about healthy eating; pregnancy challenges (cravings, eating for two, tiredness, night hunger, difficulties exercises); practical considerations (unaware of community-based service, access issues, time pressures with young family); financial difficulties; lack of motivation; happy with current weight; discomfort with being weighed; social isolation; lack of confidence to attend group sessions; lack of respect and feeling dismissed by healthcare providers; too much attention on weight in pregnancy vs. healthcare providers avoiding the issue.	Motivation (self and for baby’s health); electronic information resources; supportive peer environment; supportive / attentive healthcare providers; healthcare providers who proactively addressed weight management; input from multidisciplinary teams.
Fieldwick D, et al. 2014	New Zealand	Research paper	Hospital	Midwives	N/A	Qualitative, 12 healthcare providers	Interviews, Focus groups	Lack of knowledge about gestational weight gain, lack of guidelines and consensus for practice and referral; lack of sensitivity around the topic of weight; communication difficulties; cultural difficulties; lack of weight management resources; judgemental attitudes about weight.	Knowledge of gestational weight gain; education; increased referral, and monitoring; continuity of care; maintaining a practical focus; multidisciplinary support; improve access specialists (e/g/. dieticians); clear guidelines.
Flannery C, et al. 2018	Combination of countries	Research paper	Hospital	N/A	Pregnant women	Qualitative, 22 consumers	Interviews	Lack of physical skills; lack of information; lack of opportunity to engage in physical activity in pregnancy / hindered by work and family commitments.	Action planning, goal setting and self-monitoring; family and friends’ support.
Flannery C, et al. 2019	Combination of countries	Research paper	GP, Hospital	Midwives, consultant obstetricians and general practitioners (GPs)	N/A	Qualitative, 17 Healthcare providers	Interviews	Normalisation of obesity; challenges broaching the subject of weight; shifting the focus to the management of obstetric complications; unclear roles and responsibilities for weight management advice; broader social determinants (e.g., obesogenic food environment).	
Furness PJ, et al. 2011	UK	Research paper	Hospital, community-based service	Midwives	Pregnant women	Qualitative, 7 Healthcare providers & 6 consumers	Focus groups	Healthcare providers: Lack of Information knowledge, and skills to support weight management. Women: negative self-talk; lack of motivation; lack of social support; stigma.	Continuity of care; supportive and non-judgemental attitudes; social support and interaction with healthcare providers and other pregnant women; opportunities for peer support online and in person.
Goldstein RF, et al. 2021	Australia	Research paper	Hospital	N/A	Pregnant women	Mixed Methods, 14 interviews & 49 surveys	Surveys, Interviews	Fatigue, lack of time, lack of motivation.	Developing rapport; receiving clear advice; resources to improve health literacy; family support.
Hanley, SJ 2021	UK- England	Thesis	Community (social media recruitment)	N/A	Postpartum Women	Mixed methods, 12 interviews, 27 Questionnaires 10 PPI,	Interviews, Questionnaires, Patient and Public Involvement (PPI) work	Fatigue; lack of advice and support; work commitments; physical constraints; lack of time and finances; concerns about safety of exercise in pregnancy; pregnancy cravings; nausea.	
Heslehurst N, et al. 2011	UK	Research paper	Hospital	Midwives, obstetricians, dietitians, physiotherapists, diabetes specialist and any other members of staff with a clinical interest in maternal obesity	N/A	Qualitative, 20 Healthcare providers	Interviews, Focus groups	Lack of knowledge about obesity services; information overload; negative perceptions about the feasibility of managing obesity; limited resources; lack of clear guidelines on monitoring weight in pregnancy; difficulties in discussing obesity and risks of complications with pregnant women.	
Heslehurst N, et al. 2013	UK	Research paper	Hospital, Community based service	Midwives	N/A	Qualitative, 46 Healthcare providers	Focus groups	Uncertainty about effective communication and management; concerns of a negative impact on the midwife-woman relationship; lack of confidence in weight measurement and monitoring; patient refusal to be weighed; lack of training in communication skills, empathy, and weight management support; lack of knowledge about local support services.	
Heslehurst, N, et al. 2014	UK- but included studies from UK, US, Aus, Japan, Canada, Finland	Systematic review/meta-analysis	Hospital and community-based services	Health care professionals involved in care of pregnant women	Pregnant women	Mixed methods, 25 included studies	N/A	Lack of formal training knowledge, guidelines, skills, and confidence; social influences; lack of resources for weight assessment; avoiding difficult and sensitive conversations; health professionals own weight status, personal experiences; normalisation of obesity; lack of referral/support services; lack of time and finances.	Peer learning to promote consistent practice; women prompting health care professionals for advice and providing feedback on services used.
Heslehurst N, et al. 2017	UK	Research paper	Hospital	N/A	Pregnant women	Qualitative, 15 consumers	Interviews	Advice not tailored to consumer’s specific needs	Treatment as individuals rather than the dietitian assuming the consumer’s diet was unhealthy because of their weight; personalized services; having choice and control over changes and setting realistic and achievable goals.
Holton S, et al. 2017	Australia	Research paper	Hospital	Midwives	Pregnant women	Qualitative, 2 HEALTHCARE PROVIDERS & 17 consumers	Interviews	Healthcare providers: Lack of weight management resources to assist women; lack of formal training for midwives about caring for pregnant women with overweight and obesity.	Women: preference for midwives to discuss pregnancy weight management; support groups; smartphone weight-tracking apps and web-based resources for supporting weight management.
Johnson M, et al. 2013	USA	Systematic review/meta-analysis	General maternity settings, including community health	Healthcare professionals involved in the care of pregnant women	Pregnant women	Qualitative, 17 included studies	N/A	Women: Inconsistent use of advice and information; low health literacy and cooking skills; conflicting advice from families, partners, and healthcare providers; concerns about the safety of physical activity during pregnancy; lack of access to gym classes and outdoor physical activity facilities; pregnancy perceived to be as a socially acceptable excuse to be large; sensitivity and stigma of weight management. Healthcare providers: sensitivity of weight management; lack of knowledge about weight management; lack of continuity of care.	Appropriate access to information and advice; follow-up with the same healthcare providers; personal motivation.
Kriebs JM. 2014	USA	Discussion paper	General healthcare system	Hospital, Community based service	Pregnant women	N/A	N/A	Negative attitudes/weight stigma of healthcare providers; healthcare providers concerned about offending women; normalisation of obesity; lack of healthcare provider knowledge; lack of resources for healthcare providers; lack of healthcare provider concern about weight; low motivation of women.	Providing women with information about weight management; access to dieticians to refer women.
Leslie WS, et al. 2013	UK	Research paper	Hospital	N/A	Pregnant women	Mixed Methods, 428 consumers	Surveys	Failure to lose weight between pregnancies; lack of time to exercise; lack of access to resources and programs; caring responsibilities	Access to sport/leisure facilities; time off from work to exercise; group sessions; individual clinics.
MacAulay S, et al. 2019	UK	Research paper	GP, Community based service, hospitals and local councils	GPs, health service/ hospitals/ local council personnel	N/A	Mixed Methods, 378 surveys 14 interviews	Surveys Interviews		Consumer involvement when planning weight management programs; adequate time, personnel and finances to run programs; adhering to guidelines; increasing confidence and communication skills of midwives to support weight management; healthcare providers having the time and necessary knowledge and skills to provide weight management.
Macleod M, et al. 2013	UK	Research paper	Hospital, Community based service	Midwives	N/A	Quantitative, 78 Healthcare providers	Surveys	Perceptions that weight management is not in midwifery scope of practice; competing demands in antenatal appointments; perception that women do not want to address overweight or obesity; sensitivities about discussing weight; lack of knowledge and confidence in weight management.	Referral processes to dietitians; training for midwives to build knowledge and confidence; a bank of weight management resources (written leaflets, web resources and contacts for local community-based groups).
McCann MT, et al. 2018	UK	Research paper	Hospital	Midwives	N/A	Qualitative, 17 Healthcare providers	Interviews	Midwives lack the knowledge, expertise, confidence and resources for weight management; normalisation of obesity; lack clinical guidelines; lack of clinical leadership; midwives to recognising weight management in scope of practice; lack of referral pathways to specialists; lack of time in antenatal appointments; concern about offending women; normalisation of obesity.	
Miller M, et al. 2014	Australia	Discussion paper	GP, Hospital, Community based service,	Midwives, Doctors	Pregnant women	N/A	N/A	Healthcare provider barriers: Lack of time, remuneration and capacity to engage in weight management during antenatal appointments; absence of weight-related policy; lack of weight monitoring & identification of gestational weight gain; limited provider knowledge and inadequate advice on perinatal physical activity; perception that weight status is not important; normalisation of obesity; sensitivity to raising weight issues; weight bias; pessimism about success of weight management Service level barriers: limited demand from women; inadequate consultation time; lack of suitable support staff; lack of low-cost, local referral systems; inconsistency in weight management advice from healthcare providers and other sources; limited continuity of care between hospital, specialist, general practice and public health services.	Pregnancy as an optimal time for health providers to engage women in weight management.
Patel, C, et al. 2013	England	Research, Qualitative	Community based service	N/A	Women who declined weight management service during pregnancy	Qualitative, 15 consumers	Interviews		Midwives seen as an appropriate source of information.
Smith SA, et al. 2011	UK	Research paper	GP, Community based service	Nurses, Midwives, Doctors, Community service managers, family support workers, staff with a physical activity role, healthcare assistants, leisure centre gym staff, health visitor, teenage pregnancy support worker, project managers, leaders, and co-ordinators	N/A	Qualitative, 30 Healthcare providers	Interviews, Focus groups	Limited services available; financial constraints; environmental factors (e.g. transport); language barriers; lack of women’s understanding/ knowledge about nutrition, physical activity; lack of women’s concern about excessive weight gain in pregnancy; lack of knowledge of service providers for weight management in pregnancy; lack of appropriate specialist services, polices and guidance; financial costs to access services; funding restrictions to offer incentives for weight management; lack of management support within the organisation; lack of guidelines and evidence based information around maternal obesity; bureaucratic issues, such as working hours, reluctance to share information, and a lack of willingness of staff to change practice; time pressures of prioritising workloads; lack of resources such as sufficient manpower, capacity and suitable staff to deliver services; a lack of appropriate facilities such as the venue, location and building facilities.	Using services women already attending to support weight management; pregnancy / postnatal/breastfeeding ideal time to intervene for behaviour change and to engage women with obesity services; continuity of care; flexible access to services; multidisciplinary approach; use of appropriate language when discussing obesity; targeted services for women; group, peer and social support
Walker R, et al. 2019	Australia	Research paper	Primary Health Care Setting	General Practitioners	N/A	Qualitative, 20 Healthcare providers	Interviews	Lack of motivation in women; healthcare provider low awareness of guidelines; broader social and physical environment barriers (e.g., women’s capacity to put the advice they receive into practice).	General practitioners considering it their professional role to support women with weight management; prioritising the provision of weight management advice.
Willcox, JC, 2012	Australia	Research Paper	Rural and urban hospitals	Midwives	N/A	Qualitative, 15 Healthcare providers	Interviews	Gestational weight gain perceived as a lower priority within time limitations in antenatal appointments; perceptions that excess weight gain was not a significant health issue and women were not interested in weight management; limited education of midwives and lack of confidence in weight management; lack of weight monitoring in practice; limited resources; concern of sensitivities about discussing weight gain.	Midwives’ weight management advice; midwife education and training about weight management in pregnancy.

### Synthesis of findings

The identified barriers and enablers for weight management in healthcare settings fell under three broad themes at the individual and healthcare system level: (1) access to, and engagement with, weight management advice during pregnancy (2), challenges for providing weight management support in healthcare settings, and (3) healthcare provider confidence in providing weight management advice during pregnancy.

#### Access to, and engagement with, weight management advice during pregnancy

Women and provider perspectives

#### Barriers

##### Challenges to accessing services and engaging with weight management advice

The research highlighted that pregnant women faced numerous barriers to accessing and engaging with weight management during pregnancy. These predominantly related to practical difficulties in accessing services as identified by women, such as a lack of transport [36, 38–40], costs of services, or financial strain as a result of attending services (e.g., time away from work) [35, 40–45], lack of time [38, 43, 45, 46], and childcare [38, 40, 41, 43, 46] to attend appointments. Work [35, 38, 43, 46, 47], family commitments [45, 46, 48] and the need to travel long distances to services [36, 39, 41, 49] also acted as barriers to accessing care.

Cultural and language barriers were perceived to impact women’s engagement with services by healthcare staff [40, 42]. Additionally, reduced motivation [36, 39, 43, 45, 50–52] and a lack of family or social support [37, 44, 53] to incorporate suggested guidance for weight management were also documented as concerns by women and healthcare providers in the literature. These factors made it difficult for women to engage with weight management advice provided in their healthcare interactions.

##### Avoiding the topic of weight due to perceived weight bias and stigma

Both women and healthcare providers indicated that the topic of weight was often avoided due to its sensitive nature [36–39, 41, 42, 45, 47, 49–51, 53–57]. Women wanted to avoid judgement and blame [37, 39, 41, 43, 51, 58], while healthcare providers were concerned about distressing women and causing offence [36–39, 42, 48, 50, 51, 53, 57]. Women with overweight and obesity during pregnancy described feeling stigmatised [37, 45, 51] and reported that healthcare providers made assumptions about their weight and individual health behaviours [41, 43]. Healthcare providers recognised the presence of weight stigma and tried to raise the topic of weight gently [48], or would wait for women to raise the topic, which often didn’t occur, acting as a barrier to care [44, 56]. Midwives were especially concerned about broaching the subject of weight management as they perceived that sensitivities could impact their relationship with women [38, 53, 55, 56]. Several studies also identified that midwives were concerned about their own weight and felt they could not advise others when they themselves struggled with the same issues [36, 38, 49, 50, 53].

Women reported that healthcare providers focussed too much on the weight-related risks they may encounter during pregnancy, which left them feeling distressed, stigmatised [45, 54], judged, and blamed during their care experiences [45, 58]. This led to avoidance of discussing weight issues, presenting a barrier to engagement with weight management advice.

##### Lack of health literacy

Women not recognising overweight and obesity during pregnancy as a concern [36, 38, 45, 50, 51, 53, 56], and the high prevalence of obesity in the community leading to the ‘normalisation’ of obesity [36, 38, 42, 48, 49, 51, 55, 56] were repeatedly perceived by healthcare providers as barriers to weight management. Furthermore, women also identified a lack of understanding about how to maintain healthy behaviours during pregnancy [37, 44, 48], and highlighted concerns about the safety of engaging in activities to support weight management, such as changing diet and increasing physical activity [35, 38]. This was further compounded by women’s perception that pregnancy was considered a socially acceptable reason to gain weight [35, 37, 41, 42, 51, 54] and succumb to cravings [45]. Some women turned to various sources which were not considered evidence-based to obtain information about pregnancy weight management from websites [37, 41, 45, 54, 55], the media [37, 41], and friends experiences [41].

##### Lack of appropriate resources and consistent advice to support weight management

Women described a lack of tailored advice; they were provided either too little or too much information about maintaining their weight during pregnancy [35, 37, 38, 41, 45, 49–51, 53–55]. They were given pamphlets to take home or a referral to other services with little explanation or context for the advice [45, 58]. Conflicting weight management advice from different service providers and information sources often contributed to frustration [37, 51, 54, 58]. Women reported that advice typically centred on foods to avoid during pregnancy (e.g., due to listeriosis risk), with little focus on foods to include for a healthy diet [55] or exercises appropriate for pregnancy [48].

#### Enablers

##### Improving accessibility and acceptability of weight management support

Several studies recognised the importance of establishing weight management services closer to women’s homes to address obstacles such as transportation [38, 41, 49]. The availability of childcare at healthcare services [41] and the provision of free services (or incentives to support weight management behaviours such as gym passes) to overcome cost barriers were also described [40, 41, 55]. In addition, improving appointment availability to consider women’s other commitments, such as work, was acknowledged [40, 41, 46, 49]. Multiple studies identified that the integration of social support (e.g., peer support and family engagement) in weight management interventions helped to promote motivation and engagement with programs [36, 37, 43, 45, 51, 58].

Many studies suggested that pregnancy was an ideal time to engage women in weight management advice due to the regular contact with healthcare providers and their motivation to do what was best for the health of their babies [36, 39, 40, 49]. In addition, the provision of clear, consistent, and evidence-based information [50, 55, 57] was identified as having the potential to overcome barriers related to low health literacy and conflicting information. Women stated they needed clear advice about diet and physical activity in pregnancy that was simple to incorporate into their daily routines [41, 43, 45, 48, 49, 58]. Web based resources [45, 49, 50, 55] and weight tracking smart phone apps [48, 55] were identified as potentially beneficial sources of information or motivation for behaviour change.

Both women and midwives identified the value of midwife-led weight management care [37, 39, 42, 45, 49–51, 53, 55, 57, 58]. The ways midwives approached their care was perceived to be less threatening than other healthcare providers by women [42], as they engaged in woman-centred care and promoted the development of a trusting relationship [45]. A woman-centred approach by all healthcare providers where women were spoken to respectfully, in a non-stigmatising way was perceived to be beneficial for engaging women in weight management [39, 42, 45, 50, 53, 55]. Ensuring that gestational weight management was broached sensitively [40, 43, 45, 51, 54], discussed as a routine part of care for all pregnant women [36, 39, 45, 53, 55], and offered women choices to engage in varied approaches, such as regular weighing and provision of diet and physical activity guidance, were identified as key enablers.

###### Challenges for providing weight management support in healthcare settings

Provider perspectives

#### Barriers

##### Lack of continuity in care

Healthcare providers identified that lack of continuity in maternity care made it difficult to support weight management during pregnancy [40–42, 48]. This limited the opportunities to provide ongoing guidance and monitor women’s progress. It was suggested that following women from preconception to pregnancy and postnatal periods would be most beneficial for supporting long-term weight management [39, 54]. However, it was noted that weight management care was discontinued once the baby was born, even though women still required support in the postnatal period for long-term weight loss and reduction of associated risk factors [41, 54].

##### Lack of time and competing priorities

Healthcare providers frequently reported heavy workloads and lack of time in appointments to address weight management in pregnancy [36, 38, 42, 44, 47–50, 53, 55–57]. As a result, health issues perceived as more pressing were prioritised for discussion [38, 41, 44, 48, 53, 54, 56, 57]. Weight management was typically offered only to women with a very high BMI due to perceptions of increased pregnancy risk and limited resources available to healthcare providers [44, 48]. Healthcare providers also reported the increasing volume of information that was needed to be addressed in antenatal care appointments limiting opportunities for weight management discussions [9, 37, 38, 41, 44, 48–50, 53, 55–57].

##### Healthcare provider scope of practice, lack of care pathways and referral options

Healthcare providers, particularly midwives, did not always recognise that weight management was part of their scope of practice [48, 50, 56], limiting women’s access to advice.However, those who who did consider weight management as part of their roles, expressed difficulties in referring women to appropriate services due to poor availability, long waiting lists, and long referral to treatment times [35, 36, 39–42, 44, 48, 55–59]. This was especially prominent in public hospital-based settings where the limited capacities of on-site services (e.g., dietetics) were exhausted [36, 39, 41, 42, 48, 55–59]. General practitioners also identified difficulties in finding services to refer women to in the community [40, 44, 48]. Further challenges were identified in communication between service providers, with poor referral pathways and difficulties in sourcing multidisciplinary support for women [40, 48].

#### Enablers

##### Continuity of care in weight management

Continuity of care by healthcare providers was considered a strong enabler for encouraging ongoing weight management discussions and monitoring [36, 37, 41, 43, 50, 51, 57, 59]. This allowed a rapport and sense of trust to develop between the woman and the healthcare provider, helping to overcome sensitivities associated with weight discussions [37, 45, 51, 57]. Further, continuity was described to support consistent messaging and practices to monitor women’s progress (such as routine weighing) and helped to establish a sense of accountability [37, 43, 51, 59].

##### Provision of overweight/obesity-specific services and referral pathways

Healthcare providers identified that having a specific service for pregnant women presenting with overweight and obesity would support weight management provision [38–41, 43, 49, 51, 55]. This would allow professionals with experience in managing weight-related discussions and weight management care during pregnancy to support women in a sensitive and effective manner [40, 41, 51]. In the absence of this, clear and accessible referral pathways to ensure women receive appropriate multidisciplinary support were recommended [36, 42, 44, 53, 57].

###### Healthcare provider confidence in providing weight management advice during pregnancy

Women and provider perspectives

#### Barriers

##### Lack of formal guidelines, training, and resources for weight management in pregnancy

Studies indicated that a lack of consensus for weight management practice in pregnancy was a key issue to providing advice [37, 39, 40, 42]. Lack of guidelines [36, 39, 47–49, 53, 55–57], training and resources [36, 37, 39, 40, 42, 44, 49, 50, 53, 55, 56] for healthcare providers to effectively assess and manage weight in pregnancy were barriers to care. This lack of guidance limited the provision of weight management advice and identification of appropriate services to refer to [36, 39, 40].

#### Enablers

##### Provision of healthcare provider education, guidelines, and tools for standardised practice

Numerous studies identified that providing specific education to staff engaged in antenatal care was needed to support weight management in pregnancy [36, 39, 44, 47, 49, 53, 57]. Guidelines and tools for assessment and practice to standardise care and ensure all eligible women were receiving recommended approaches were needed [36, 42, 44, 47, 49, 50, 57]. Overcoming sensitivities, with education to support discussions about overweight in pregnancy was identified as an important enabler for the provision of weight management care [36, 38, 42, 48, 49, 53, 57]. Developing a shared language to frame conversations and avoid stigmatisation across all healthcare professions involved in weight management was also highlighted [36, 40, 48, 54].

## Discussion

The aim of this rapid scoping review was to collate the available literature to synthesise the key barriers and enablers to weight management in healthcare settings for pregnant women living with overweight or obesity in high income countries. Barriers and enablers were identified for pregnant women, healthcare providers, and at the health service level, indicating that multi-level strategies are needed to improve access to, and engagement with, the delivery of weight management care among pregnant women.

Pregnant women described challenges in accessing services that were both practical and personal in nature. To overcome these, they reported a preference for local services that considered their holistic needs (such as availability of childcare) to allow them to engage with weight management services during pregnancy effectively. Furthermore, ensuring healthcare providers recognised the sensitivity and stigma surrounding overweight and obesity was described. Making discussions about gestational weight gain a routine part of care for all pregnant women and delivering clear and consistent messages were considered key enablers for engaging with weight management advice.

Research has shown that pregnant women with overweight and obesity are more likely to report negative experiences of care when compared to pregnant women who had average or lower BMI [60–61]. This may be due to the tendency for maternity care staff to perceive pregnant women with overweight or obesity less positively and less likely to be engaging in healthful behaviours than pregnant women who were not considered overweight [60]. As the current scoping review identified, pregnant women living with overweight or obesity perceived a sense of judgement and blame from their healthcare providers, which acted as a barrier to engaging with weight management care. Weight stigma, which includes stereotyping, social devaluation, and alienation of women during pregnancy [62], has emerged as a concerning issue within the realm of maternity care [63, 64]. Pregnancy is a unique time when body weight can come into focus. Moreover, in society, pregnancy is often idealised as a time of health and vitality, and societal emphasis on thinness and body image ideals may lead to judgment and stigmatisation of women who do not conform to these standards during pregnancy [64]. Healthcare providers who discuss weight in a stigmatising manner may cause pregnant women to internalise the stigma, impacting their emotional and health-related behaviours, leading to greater weight gain and the development of health issues in later life for both mother and infant [65, 66].

Weight stigma among healthcare providers can be attributed to a complex interplay of societal, cultural, and individual factors [67]. In turn, these implicit biases against people living with overweight or obesity may lead to suboptimal communication, engagement, and care provision [67]. Further, this rapid review highlighted that many healthcare providers avoided discussing weight with women during pregnancy because they were conscious of the potential for weight stigmatisation and its consequences [38, 50, 53, 55, 56]. A scoping review by Dieterich and Demirci [68] identified that weight stigma impacted the delivery of weight related communication in obstetric settings. This may be due to a lack of adequate training for healthcare providers to address weight-related issues sensitively and effectively [36, 38, 42, 48, 49, 53, 57].

Given the importance of body weight and weight change in pregnancy, further work is required to foster a supportive and non-judgemental healthcare environment [63, 64]. Addressing weight-related stigma in pregnancy requires a multi-faceted approach that involves healthcare professionals, policymakers, and the community [60, 61]. Comprehensive education and training programs for healthcare professionals, co-designed with women and families, are crucial to raise awareness about weight bias, promote empathetic communication, and ensure woman-centred care is provided [58, 61]. Collaborative efforts between healthcare organisations, advocacy groups, and policymakers can help reduce societal pressures related to pregnancy and body image ideals. Implementing guidelines that focus on respectful and non-judgmental communication, both in healthcare and the wider society, could contribute to a more supportive environment [69].

Continuity of care in weight management was identified as an important element to ensure pregnant women develop relationships and a sense of trust with their care providers to overcome negative perceptions and stigma [37, 45, 57]. All of these aspects fit in with a woman-centred care approach, which is the tenet of midwifery practice [70]. Pregnant women’s appreciation of a woman-centred approach could be the reason why midwifery-led weight management was identified as a preferred method for receiving care [37, 39, 42, 45, 49–51, 53, 55, 57, 58]. However, midwifery staff did not always identify weight management as within their scope of practice or felt they lacked the appropriate knowledge and training to give such advice [36, 72]. A recent pilot trial of a training package to support weight management guideline implementation in midwifery care showed improvements in midwives self-efficacy to provide weight management [73]. More effectively preparing midwives to provide weight management support is thus an important strategy for improving care and overcoming weight stigma [72]. Furthermore, as weight management is most effective with multidisciplinary practice, supporting all healthcare clinicians providing care to pregnant women to develop skills in non-stigmatising, woman-centred care through appropriate training is also likely to improve engagement with weight management [74].

The lack of clear health service guidelines and consistency in practice to support weight management in pregnancy was repeatedly identified by healthcare staff. A recent clinical guideline review of National Health Service Trusts in England demonstrated that guidelines primarily focussed on measuring weight at booking and informing women of risks associated with obesity in pregnancy [75]. The guidelines varied in advice for assessing and monitoring weight, and pathways for referral were unclear, indicating a lack of guidance and standardisation for appropriate practice [75]. Similar ambiguity and focus on risk is reflected in guidelines from other high-income countries such as Australia [76] and the United States of America [77]. Emphasising risk has been an established barrier to pregnant women’s engagement with weight management care [45, 54]. More effectively conveying risk using an individualised and woman-centred approach with tailored weight management strategies is likely to promote engagement [75]. Supporting healthcare services to implement guidelines and policies for practice and referral is also needed to establish a consensus for weight management care in pregnancy across healthcare systems [74].

Alongside guideline development, it is essential for healthcare systems to invest in healthcare provider training and education on stigma-free weight management care in pregnancy. The review highlighted that skills in communicating about overweight, obesity, and weight management during pregnancy are a clear need for healthcare providers. It appears that training that focuses on how to communicate, rather than what to communicate, is necessary and should be informed by meaningful stakeholder engagement that is inclusive of pregnant women’s voices [74]. Training alone is unlikely to be adequate without implementing supportive policies, such as allocating sufficient time for antenatal appointments, and having sufficient referral pathways and options for pregnant women to access multidisciplinary care [39, 78]. This reflects the previously stated need for multi-level strategies to improve access to, engagement with, and delivery of weight management care among pregnant women.

### Strengths and limitations.

All the included studies presented results regarding barriers and/or enablers for weight management in healthcare settings providing care to women during pregnancy. The identified literature included a range of publication types, however, as this was a rapid review, limitations of the search may have resulted in missing relevant publications. Furthermore, as this was a sub-analysis of a larger review, the initial title and abstract screening may have missed relevant publications due to the original focus. It has, however, been demonstrated that the barriers and enablers to weight management in pregnancy were similar in many of the papers, providing a good scoping of the key issues. Consistent with scoping review methodology [28], a quality appraisal was not conducted, therefore, low-quality studies may have been included. Furthermore, the review focused on English-speaking high-income countries, limiting the generalisability of the results beyond these settings. There was also a lack of literature on priority populations such as First Nations people and socially disadvantaged groups, identifying gaps requiring future focus. Despite these limitations, this rapid scoping review provides an overview of evidence describing key barriers and enablers for weight management care to pregnant women living with overweight or obesity.

## Conclusion

Pregnant women living with overweight or obesity experience a number of individual, provider, and health service barriers and enablers to effective weight management. Findings of this rapid review suggest that multi-level strategies are needed to ensure pregnant women have access to weight management care, which is stigma-free, easily accessible, tailored to their individual needs, and which fosters positive relationships with healthcare providers. In addition, ensuring optimal ongoing education and training for providers to ensure sensitive communication and confidence in engaging in weight-related discussions was highlighted. Furthermore, support for implementing clinical guidelines to ensure consistency in weight management practice through the antenatal period is warranted.

## Data Availability

Literature analysed in the study can be sourced by referring to citations included in the results section in the paper.
